# Improvement of Dosimetry Planning in 3D Conformal Radiotherapy for Breast Cancer With Lymph Nodes Using the High‐Tangential Tilted‐Supraclavicular Technique

**DOI:** 10.1155/ijbc/2650553

**Published:** 2026-04-15

**Authors:** Mazen Moussallem

**Affiliations:** ^1^ Research and Development Department, Healthy Innovations, Rachiine, Zgharta, Lebanon; ^2^ Radiotherapy Department, Centre de Traitement Médical du nord, Zgharta, Lebanon; ^3^ Medical Imaging Department, Holy Family University, Batroun, Lebanon, holyfamily.edu

**Keywords:** 3DCRT, breast cancer, dosimetry planning, lymph nodes

## Abstract

**Purpose:**

Despite the emergence of advanced radiotherapy techniques such as volumetric modulated arc therapy (VMAT), three‐dimensional conformal radiotherapy (3DCRT) continues to be used due to its advantages in treating breast cancer. This study aimed to optimize the 3DCRT technique for breast cancer cases that require lymph node irradiation.

**Methods and Materials:**

A total of 100 consecutive patients were included. For the first 50 patients, standard (ST) 3DCRT dosimetry plans were prepared. For the remaining 50 patients, plans were generated using an optimized High‐tangential Tilted‐supraclavicular (HT) technique, which consists of tilting the supraclavicular beam to reduce ipsilateral lung irradiation; raising the tangential fields superiorly to enhance coverage of axillary levels I and II; and intentionally creating an internal overlap between the supraclavicular and tangential fields. This overlap was resolved using the field‐in‐field (FIF) technique, primarily applied to the supraclavicular field, which is the main contributor to ipsilateral lung dose in this region.

**Results:**

The HT technique demonstrated promising results compared with the ST technique and other published 3DCRT methods. It achieved improved coverage of axillary levels I and II while reducing the dose to the ipsilateral lung. However, further adjustments are needed to enhance coverage of axillary level III, the supraclavicular region, and internal mammary nodes (IMN). These include the following: completing targets contouring prior to dosimetry planning to enable precise field adjustments; relaxing the 2 cm constraint on ipsilateral lung irradiation by tangential fields when IMN is included; relaxing the maximum dose constraint for the supraclavicular and IMN fields; and using a fully electron‐based field instead of a mixed electron‐photon technique for IMN dedicated field.

**Conclusions:**

The HT technique was quantitatively validated, and a modified version was proposed and qualitatively assessed. This new version may be considered for clinical use, and comparison with VMAT in a larger patient cohort is recommended.

## 1. Introduction

Approximately one in five individuals worldwide will develop cancer during their lifetime, with about one in nine men and one in 12 women dying from the disease [1]. Female breast cancer was the second most frequently diagnosed malignancy globally. Among women, breast cancer was the most commonly diagnosed cancer and the leading cause of cancer‐related death, ranking fourth overall in global cancer mortality.

Radiation therapy (RT), alongside surgery and chemotherapy, including targeted therapy and immunotherapy, plays a vital role in the multidisciplinary treatment of breast cancer. In the United States, RT was incorporated into the treatment regimen for at least 51% of women with early‐stage breast cancer, 26% of those with Stage III disease, and 63% of those with Stage IV disease [2]. Furthermore, the inclusion of regional lymph nodes in RT for early‐stage breast cancer has been shown to significantly reduce mortality [3, 4]. However, expanding the irradiation field to cover regional nodes inevitably increases the volume of normal tissue exposed to radiation, thereby elevating the risk of toxicity to organs at risk (OARs), particularly the lungs [5].

Minimizing radiation‐induced toxicity to OARs remains a major priority in modern radiotherapy [6]. To limit radiation exposure to the heart and lungs during breast cancer treatment, breathing‐adapted techniques such as deep inspiration breath hold (DIBH) and continuous positive airway pressure (CPAP), a newer alternative to DIBH, have been adopted [7]. However, while advanced technologies like intensity‐modulated radiotherapy (IMRT) and volumetric modulated arc therapy (VMAT) offer improved target coverage, they often result in increased low‐dose exposure to surrounding OARs [8]. This elevated low‐dose exposure has been associated with a higher risk of secondary malignancies in VMAT and IMRT compared with three‐dimensional conformal radiotherapy (3DCRT) [9].

For breast cancer, 3DCRT may offer practical advantages over IMRT and VMAT. For example, 3DCRT enables faster treatment delivery [10], which benefits patients undergoing DIBH by reducing breath‐hold durations. Moreover, IMRT or VMAT planning can be highly dependent on the planner’s expertise and demands special attention to the extension of the planning target volume (PTV) toward the skin surface, akin to static fields with a large skin flash in 3DCRT. This usually necessitates specialized planning procedures in IMRT or VMAT, for example, creating an initial dosimetry plan with a bolus on the skin to allow the multi leaf collimator (MLC) to open toward the PTV in the direction of the skin [11]. For treatment delivery, the same plan is then recalculated after removing the bolus, while maintaining the MLC configuration. The importance of these procedures may be underestimated, and they may not be feasible for a large number of dosimetrists. In addition, in the absence of online adaptive radiotherapy, significant breast deformation, greater than 1 cm near the PTV extension, may require IMRT or VMAT replanning to ensure treatment accuracy [11].

To enhance nodal coverage in 3DCRT, high tangential fields (HTF) have been routinely employed to irradiate the axillary lymph nodes [12–14]. Separately, Yang et al. [15] proposed a technique to reduce lung volume and dose during supraclavicular irradiation for breast cancer. This method is based on the observation that the angulation of the breast board used for tangential breast irradiation inadvertently includes more normal lung tissue in the supraclavicular field. To address this, their approach involved rotating the treatment couch by 90° and tilting the supraclavicular field (TSF) to maintain a perpendicular beam angle relative to the breast board rather than the couch surface, thereby sparing more normal lung tissue.

Inspired by the HTF and TSF techniques, a new method, referred to as the High‐tangential Tilted‐supraclavicular (HT) technique, was developed and evaluated in the present study. This approach combines both strategies with the aim of achieving adequate lymph node coverage while reducing lung dose compared to the standard (ST) 3DCRT technique.

## 2. Methods and Materials

Computed tomography (CT) scans from 100 consecutive patients treated with radiotherapy for breast cancer involving lymph nodes were included in this study. During CT acquisition, patients were immobilized in the supine position on a CIVCO radiotherapy breast board, with both arms and hands resting on supports positioned around the head. For the first 50 CT scans, used in the present study for planning with the ST 3DCRT technique, the breast board inclination was set to 12.5°. In contrast, for the remaining 50 CT scans, used for planning with the HT 3DCRT technique, the breast board inclination was set to 0°. Despite this difference in breast board inclination, the two patient groups are comparable. When the thorax is used as the anatomical reference, the thoracic region, OARs, and the breast‐related lymph node areas maintain the same relative positions, regardless of the breast board angle (0° vs. 12.5°). Changing the breast board angle results only in a global rigid transformation of the thorax within the scanner coordinate system, without altering internal anatomy or the spatial relationships among these structures. In practical terms, the thorax’s anatomy remains unchanged, and only the orientation of the upper body in space is modified. Since our analysis focuses exclusively on the thorax and upper nodal regions, and does not include the lower body (abdomen and pelvis), the two patient groups can therefore be directly compared. In all patients, the head was turned to the contralateral side. CT scans were acquired during free breathing (FB), using a slice thickness of 2.5 or 3 mm. A commercial treatment planning system (TPS), Prowess Panther version 5.2 (Prowess, Inc., CA, United States), commissioned for a Siemens Artiste linear accelerator (Munich, Germany), was used to generate the dosimetry plans for the present study.

Each of the two groups, ST and HT, was further subdivided into subgroups based on: (1) the side of treatment (right [R] or left [L]); (2) whether the internal mammary nodes (IMN) were included using a dedicated direct field (+ IMN); (3) cases where the IMN was not included, but a complementary field (+ C), similar to the dedicated direct IMN field, was used to cover the entire medial breast or chest wall when the tangential fields alone could not provide adequate coverage; and (4) cases where the IMN was included within the tangential field without a dedicated direct IMN field, by using wide tangential (WT) fields [16, 17].

The clinical treatment prescriptions associated with the CT scans were used to prepare the dosimetric plans in this study. They were as follows: 50 Gy in 25 fractions for breasts or chest walls; 46 Gy in 23 fractions for IMN or C regions (if included); and 45 Gy in 25 fractions for the remaining lymph node regions. Boost volumes delivered in subsequent phases were not included in this study.

### 2.1. ST 3DCRT Technique

The ST technique used in this study was based on a standardized beam arrangement employing a multi‐isocentric approach, inspired by the technique described by Fontanilla et al. [18], but modified to use for the IMN or C fields (when included) a combination of electron and photon beams, rather than electrons alone, as proposed by Goyal et al. [19] This modification aimed to reduce electron‐related skin toxicity. Consequently, dosimetry planning for the IMN or C regions was performed using a combination of 13 electron fractions (56.5% of the 23 fractions) and 10 photon fractions (43.5% of the 23 fractions) with beam angles inclined between 10° and 15°.

These IMN or C beams were matched at the skin surface with medial tangential photon beam. Medial and lateral tangential photon beams were oriented to limit irradiation to no more than 2 cm of the ipsilateral lung and 0.5 cm of the heart, based on the beam’s eye view. The medial tangential field was defined first; the lateral tangential field was then created by mirroring and opposing the medial field, with gantry rotation adjusted to ensure that the posterior edges of both beams were non‐divergent and matched each other. Dose homogeneity in these tangential fields was improved using a combination of dynamic wedges and the field‐in‐field (FIF) technique.

The IMN or C fields, if included, as well as the medial tangential field were matched at the skin to a supraclavicular photon beam angled 10° away from the spine. When necessary, for medial and lateral tangential beams, the treatment couch was rotated by approximately 5° to prevent beam divergence and maintain skin‐level matching between medial tangential and supraclavicular fields. The inferior border of the supraclavicular field was aligned with the lower edge of the clavicular head, while the superior border extended to the upper part of the clavicle, but not as high as the cricoid level, as described by Arbab et al. [16]. However, in accordance with Arbab’s recommendations, the MLCs were shaped to shield the humeral head laterally and the spinal pedicles medially. The larynx and trachea were also protected using MLCs.

For the IMN and C fields, prescription points were optimized to achieve optimal target coverage while not exceeding 108% of the prescribed 46 Gy, minimizing lung dose and keeping the mean heart dose below 5 Gy in the composite plan sum with all remaining fields. For medial and lateral tangential beams, a maximum dose of 108% of the prescribed 50 Gy was allowed; however, in skin fold regions, this limit was reduced to 105%. For the supraclavicular field, the prescription point was selected to ensure the maximum dose did not exceed 108% of the prescribed 45 Gy.

Finally, field matching was planned to ensure that the global maximum dose in the composite plan sum remained below 55 Gy, corresponding to 110% of the prescribed 50 Gy.

### 2.2. HT 3DCRT Technique

The HT technique is an optimization of the ST technique. However, in the present study, this optimization was not applied to the IMN or C fields, which remained unchanged in the HT technique. In the current section, the modifications introduced in the HT technique are described; any components not mentioned can be assumed the same as in the ST technique.

As mentioned above in the Introduction section, the supraclavicular field modification was inspired by the method described by Yang et al. [15], which involves rotating the treatment couch by 90° and tilting the supraclavicular field to maintain a beam angle perpendicular to the breast board rather than to the couch surface, thereby aligning the beam perpendicularly to the posterior thoracic (back bone) surface. Consequently, in the present study, to avoid couch rotation during treatment while achieving the same beam orientation relative to patient anatomy, dosimetric plans were performed on CT scans with the breast board inclination set to 0°.

To illustrate the step‐by‐step implementation of the HT technique, an example of a dosimetry plan including a dedicated direct IMN field is presented in Figure 1. Figure 1a shows the supraclavicular field, planned using the same procedures described in the ST 3DCRT technique section, but performed on CT scans acquired with the breast board inclination set to 0°. MLC projection of the supraclavicular field on the patient’s skin is indicated in cyan in Figure 1b,c. After contouring the IMN on the CT slices, an IMN field is added to cover the IMN region (highlighted in pink in Figure 1b,c). Subsequently, the medial and lateral tangential fields (shown in red in Figure 1b,c) are placed. The collimators angles are then adjusted so that the lateral edge of the IMN field was approximately aligned on the skin with the medial edge of the medial tangential field. Finally, the IMN field position was adjusted to ensure an overlap of at least 0 mm at the skin surface with the supraclavicular field, and an overlap of 5–10 mm with the medial tangential field (Figure 1b). Because of the unavailability of a Surface Guided Radiotherapy (SGRT) device, a positioning field (Figure 1d) was created based on the tangential fields’ isocenter to verify patient positioning using the field light before treatment.

Figure 1Example of dosimetry planning using the HT technique on CT scans acquired with 0° breast board inclination. (a) Beam’s eye view of the supraclavicular field. (b, c) MLC projections on the patient’s skin. (d) Positioning field used for patient setup verification. (e) Beam’s eye view of the medial tangential field showing the matched superior border with the supraclavicular field, represented by an interpolated straight line based on the attached colored dots. (f, g) Superior edge of the lateral tangential field aligned to pass through the lateral‐inferior corner of the supraclavicular field, indicated by pink and dark green dots. (h, i) FIF subfields added to the medial and lateral tangential fields to shield hotspots (blue). (j, k) Two supraclavicular FIF subfields created to reduce doses exceeding 60 Gy. (l, m) MLC adjustments in tangential subfields further reduce hotspots below 55 Gy. (n, o) Final MLC refinements in medial tangential and supraclavicular subfields in the composite plan including the IMN field. (p–r) Resulting total dose distribution shown in axial, coronal, and sagittal views.

**Figure:**
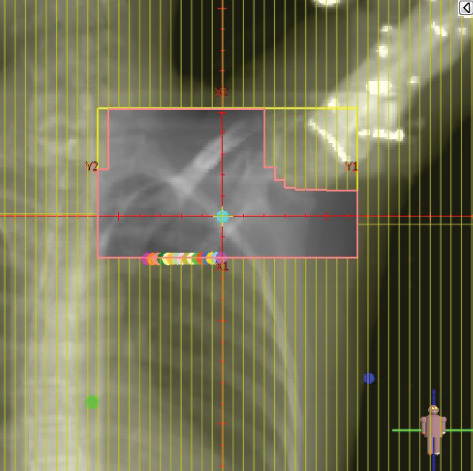
(a)

**Figure:**
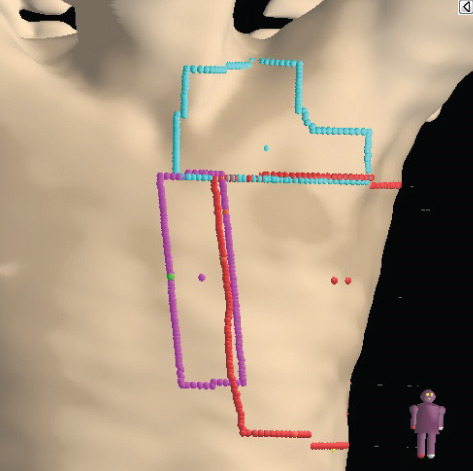
(b)

**Figure:**
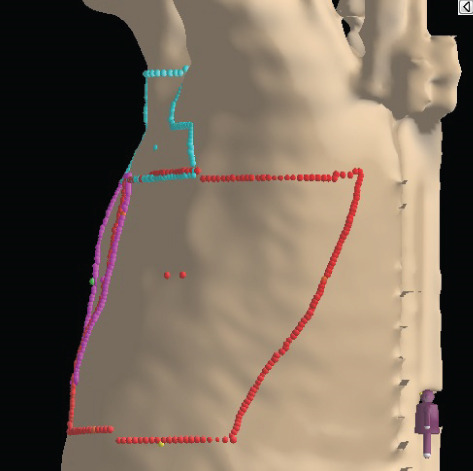
(c)

**Figure:**
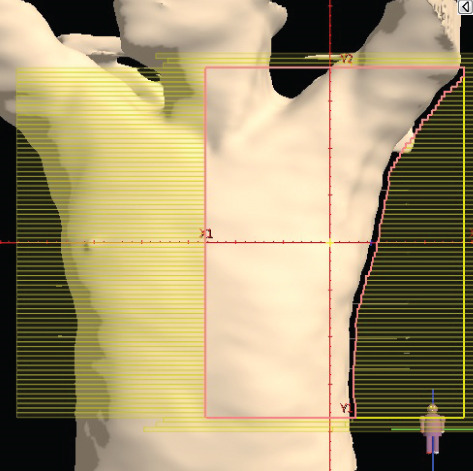
(d)

**Figure:**
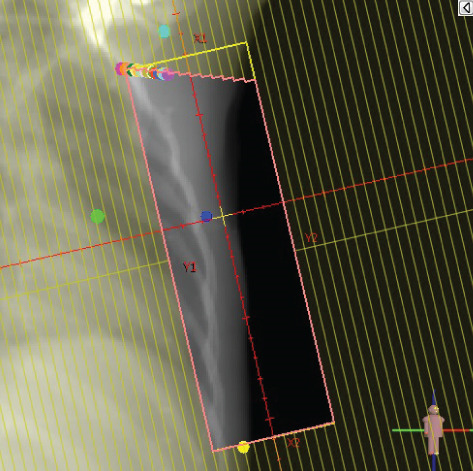
(e)

**Figure:**
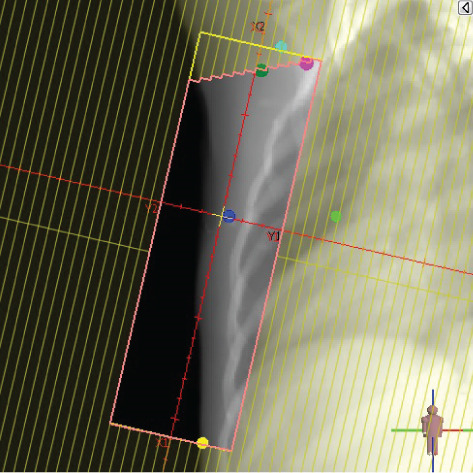
(f)

**Figure:**
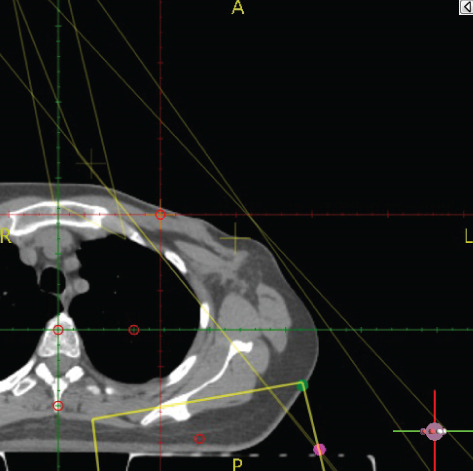
(g)

**Figure:**
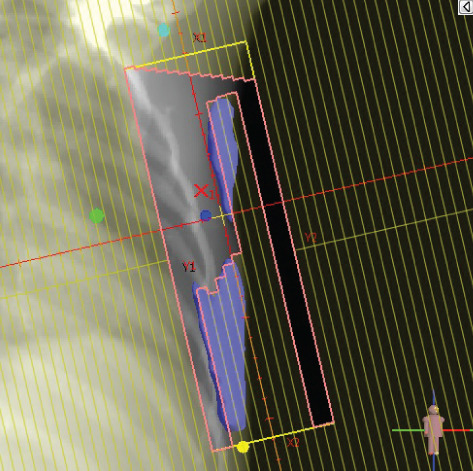
(h)

**Figure:**
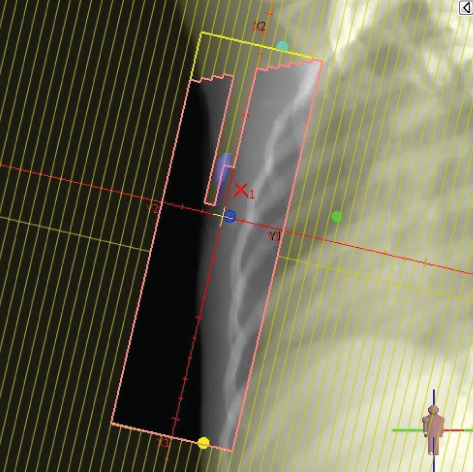
(i)

**Figure:**
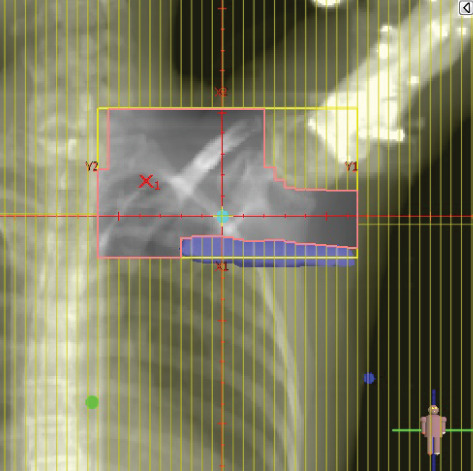
(j)

**Figure:**
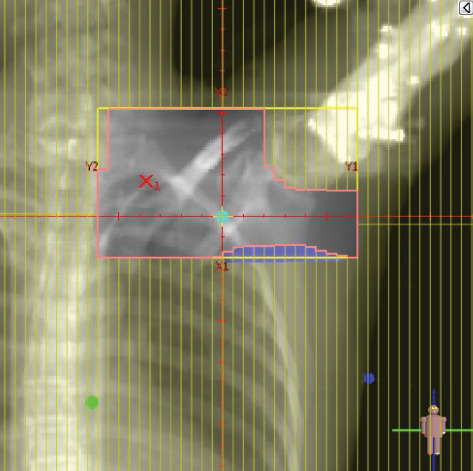
(k)

**Figure:**
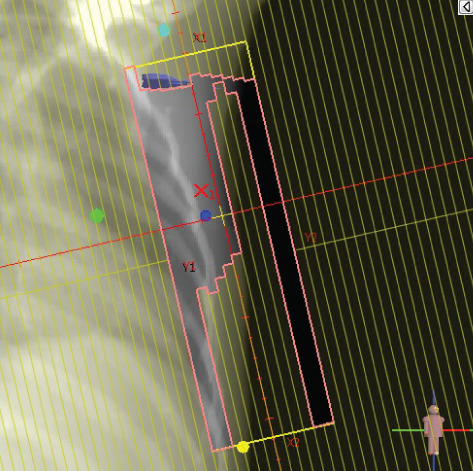
(l)

**Figure:**
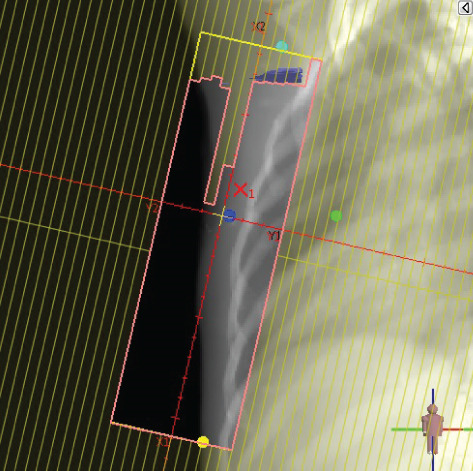
(m)

**Figure:**
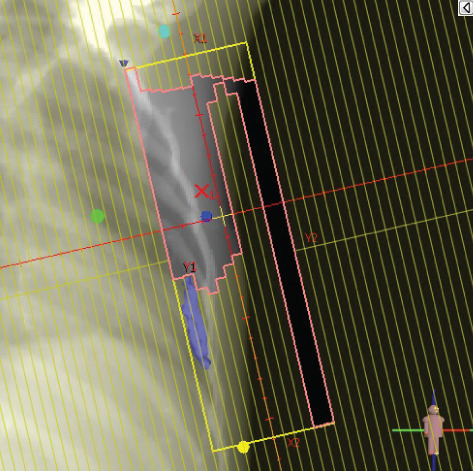
(n)

**Figure:**
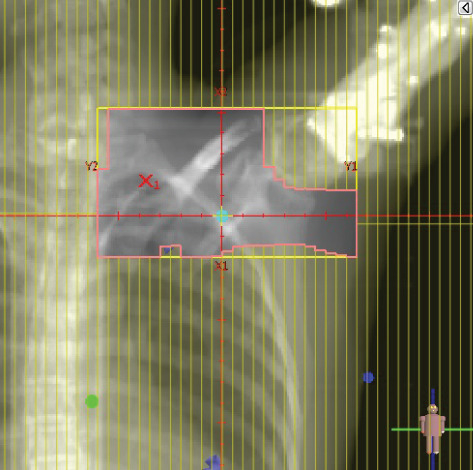
(o)

**Figure:**
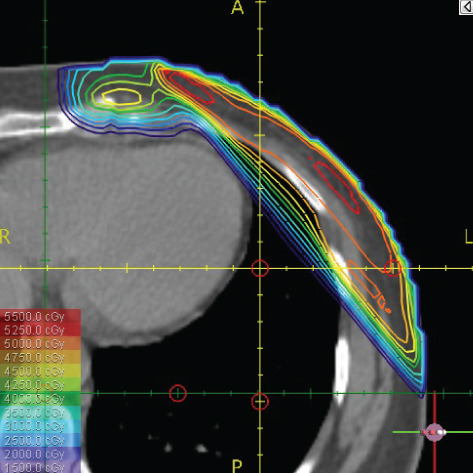
(p)

**Figure:**
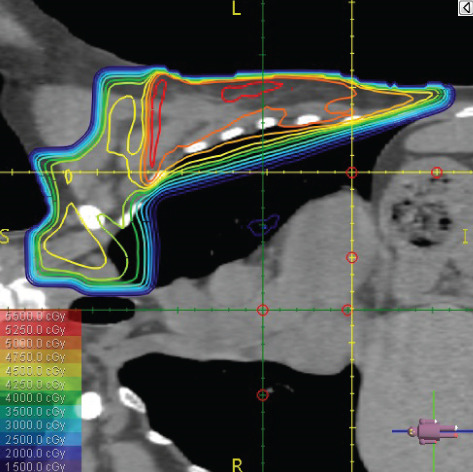
(q)

**Figure:**
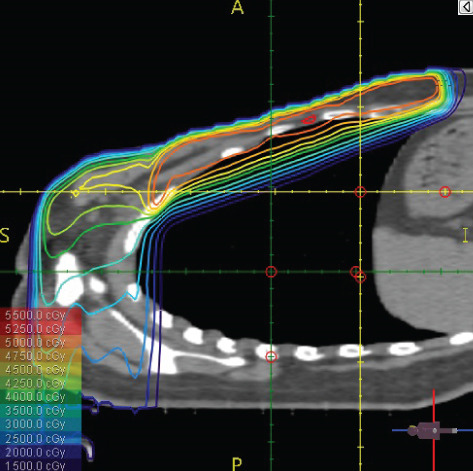
(r)

In contrast to the ST technique, no couch rotation was applied for the medial and lateral tangential beams. Their superior borders were extended despite the divergence created by the multi‐isocentric technique, which can result in internal overlap between the tangential fields and the supraclavicular field. This overlap is very important for the HT technique because it helps to deliver dose to these regions using tangential fields rather than the supraclavicular field, thereby minimizing the dose to the ipsilateral lung. The medial tangential field matched the supraclavicular field on the skin only over the first few centimeters on the medial side. This matched region is clearly visible in Figure 1b and is represented by skin‐colored attached dots in Figure 1a,e. In this matched area, the colored attached dots form a straight line in Figure 1e, which represents the beam’s eye view of the medial tangential beam. However, if extending these colored attached dots along the inferior border of the supraclavicular field on the skin does not produce a straight line in Figure 1e. Therefore, the superior edge of the medial tangential beam was interpolated as a straight line based on the matched portion with the supraclavicular field, as represented by the interpolation of colored attached dotes in Figure 1e. On the other hand, the superior edge of the lateral tangential field was adjusted to form a straight line (Figure 1f) that passes through the lateral inferior corner of the supraclavicular field, represented by the dark green and pink dots in Figure 1f,g.

**Figure 2:**
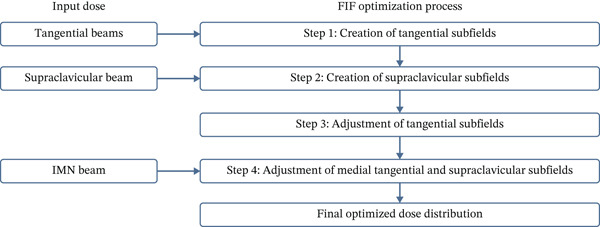
Schematic representation of the sequential FIF optimization process in the HT technique. The diagram illustrates the stepwise integration of tangential, supraclavicular, and IMN beam doses. Initially, tangential subfields are created to reduce medial and lateral hotspots. Subsequently, supraclavicular subfields are added to control the dose at beam junction regions. After incorporating the supraclavicular dose, tangential subfields are further adjusted. Finally, following the addition of the IMN beam, medial tangential, and supraclavicular subfields are refined to achieve the final optimized dose distribution.

In the HT technique, the FIF method was used to improve dose homogeneity and reduce hotspots. The process was performed in sequential steps, as schematized in Figure 2:

1. Creation of tangential subfields: As in the ST technique, in addition to using dynamic wedges, subfields were created for the medial and lateral tangential beams to shield hotspots, indicated by the blue regions in the medial and lateral subfields (Figure 1h,i, respectively).
2. Creation of supraclavicular subfields: To avoid excessive dose at the junction with the tangential fields, two supraclavicular subfields were created (Figure 1j,k) to shield regions receiving more than 60 Gy, while ensuring that the original supraclavicular field delivered at least 40% of the prescribed supraclavicular dose.
3. Adjustment of tangential subfields after adding the supraclavicular dose: The MLCs of the previously created tangential subfields (Figure 1h,i) were modified on the supraclavicular side to further reduce hotspots to below 55 Gy (Figure 1l,m).
4. Final refinement after adding the IMN dose: In the final composite plan including all fields (tangential, supraclavicular, and IMN fields), the MLCs in the medial tangential and supraclavicular subfields were further refined on the IMN side to maintain hotspots below 55 Gy (Figure 1n,o).

The resulting dose distribution is illustrated in Figures 1p, 1q, and 1r (axial, coronal, and sagittal views).

### 2.3. RTOG‐Based Contour Delineation and Dosimetric Metrics

Prior to dosimetry planning, only the OARs and IMNs, when included in the prescription, were delineated following an internal departmental contouring protocol. However, after dosimetry planning, for evaluation purposes, all volumes used in the statistical analyses, including IMNs, were delineated by the same person according to the Radiation Therapy Oncology Group (RTOG) guidelines [20], except for the ipsilateral lung, for which the original pre‐dosimetry contour was retained due to the unavailability of specific RTOG guidelines for this OAR. Consequently, the following structures were delineated: ipsilateral lung; heart; clinical target volume (CTV) of the breast or chest wall (CW); CTV of the breast or CW minus 5 mm from the skin surface; supraclavicular region; axillary levels I, II, and III; and IMNs. Based on the objectives of the HT technique, Table 1 provides a summary of the intended goals for each area, highlighting the procedures used to achieve these goals that could not be accomplished using the ST technique.

**Table 1:** Summary of the intended goals of the HT technique compared with the ST technique for each area and the corresponding procedures used to achieve them.

Area	HT intended goals	Procedures
Ipsilateral lung	Further minimizing the ipsilateral lung dose	Avoiding lung exposure by tilting the supraclavicular beam
Heart	No intended goal	No additional procedures were used compared with the ST
CTV Breast or CW	Improve dose coverage in the superior region at the junction with the supraclavicular beam	Using HTF technique and creating a deep internal overlap between the supraclavicular and tangential fields
(CTV Breast or CW) −5 mm from skin surface		
Supraclavicular	No intended goal	No additional procedures were used compared with the ST
Axillary Level I	Improve dose coverage	Using HTF technique and creating a deep internal overlap between the supraclavicular and tangential fields
Axillary Level II		
Axillary Level III	No intended goal	No additional procedures were used compared with the ST
IMN		

Based on the resulting composite dosimetry plan, the following dosimetric parameters were recorded for each patient: mean dose (Gy), V_5Gy_ (%), V_20Gy_ (%), and V_30Gy_ (%) for the ipsilateral lung; mean dose (Gy), V_20Gy_ (%), V_30Gy_ (%), and V_35Gy_ (%) for the heart; and mean dose (Gy), D_95%_ (Gy), D_90%_ (Gy), V_47.5Gy_ (%), V_45Gy_ (%), V_42.75Gy_ (%), and V_40.5Gy_ (%) for target volumes. Here, the mean dose (Gy) represents the average radiation dose received by a specific volume; V_xGy_ (%) denotes the percentage of a structure’s volume receiving at least x Gy; and D_x%_ (Gy) indicates the minimum dose received by x% of the volume.

For each subgroup of dosimetry plans corresponding to patients with the same side and prescription, dosimetric parameters from the ST and HT techniques were compared using a *t* test (performed in R version 4.2.0). *p* values less than 0.05 were considered statistically significant.

## 3. Results

### 3.1. Demographic Data

Table 2, which presents the distribution of the 100 consecutive patients included in the present study, shows that 57 were treated for all regional nodes excluding the IMN, and 43 were treated including the IMN. Among these 43 patients, according to the Methods and Materials section guidelines, which stipulate that no more than 2 cm of the ipsilateral lung should be irradiated with tangential fields, the inclusion of the IMN in WT fields was feasible in only one case. Consequently, this single case represents 2.3% of the patients for whom IMN irradiation was required. However, this patient could not be included in the statistical analysis due to the very small sample size. Dosimetric data for this patient correspond to patient number 57 in Table S1, which includes dosimetric data for all 100 patients. This patient demonstrated the highest IMN coverage among the 43 patients for whom the IMN was intended to be treated. The coverage metrics were as follows: 46.30 Gy (mean dose), 37.60 Gy (D_95%_), 42.00 Gy (D_90%_), 50.00% (V_47.5Gy_), 79.20% (V_45Gy_), 87.50% (V_42.75Gy_), and 93.10% (V_40.5Gy_). Additionally, this patient had the lowest ipsilateral lung V5Gy of all 43 patients, measured at 28.90%. Among the 20 left‐sided cases that included IMN treatment, this patient also recorded the lowest mean heart dose, at 1.10 Gy.

**Table 2:** Distribution of the 100 patients according to IMN inclusion or exclusion; treatment side ([R] or [L]); method (adding a complementary C field when tangential fields alone cannot cover the entire breast or CW, and including IMN in wide tangents when feasible without irradiating more than 2 cm of lung); and technique (ST or HT).

	All nodal regions except IMN				All nodal regions including IMN			
	R	L	R + C	L + C	R + IMN	L + IMN	R WT	L WT
ST technique	9	12	3	4	11	11	0	0
HT technique	8	10	5	6	12	8	0	1
ST + HT techniques	17	22	8	10	23	19	0	1
	57				43			

### 3.2. Ipsilateral Lung

The HT technique demonstrated a clear and significant improvement in reducing the dose to the ipsilateral lung compared with the ST technique, as indicated by the green cells in the ipsilateral lung section of Table 3. A visual representation of the data distribution is provided by the corresponding boxplot shown in Figure 3a. For right‐sided breast or CW plans that included all nodal regions except the IMN, represented by the “(HT R) ‐ (ST R)” column, improvements were observed with reductions in all dosimetric constraint values as follows: 2.35 Gy (6.98 vs. 9.33), 7.53% (26.04 vs. 33.57), 5.89% (13.44 vs. 19.33), and 5.45% (8.83 vs. 14.28) for mean dose, V_5Gy_, V_20Gy_, and V_30Gy_, respectively, with values in parentheses corresponding to HT and ST in that order, here and throughout. However, for left‐sided breast or CW plans that included all nodal regions except the IMN, represented by the “(HT L) ‐ (ST L)” column, a significant improvement was observed only for V_30Gy_: 3.86% (11.12 vs. 14.98). For right‐sided breast or CW plans with a C field, represented by the “(HT R + C) ‐ (ST R + C)” column, as well as for left‐sided breast or CW plans with a C field, represented by the “(HT L + C) ‐ (ST L + C)” column, there were no significant differences between the HT and ST techniques regarding the ipsilateral lung dose. However, when the IMN field was included, clear and significant reductions in ipsilateral lung dose values were observed for all constraints except for right‐sided V_5Gy_. These improvements were as follows: 2.47 Gy (12.12 vs. 14.59), 6.88% (17.13 vs. 24.01), and 5.22% (9.63 vs. 14.85) for mean dose, V_20Gy_, and V_30Gy_, respectively, on the right side; and 3.29 Gy (9.43 vs. 12.72), 14.18% (54.76 vs. 68.94), 8.54% (14.60 vs. 23.14), and 6.41% (8.80 vs. 15.21) for mean dose, V_5Gy_, V_20Gy_, and V_30Gy_, respectively, on the left side.

**Table 3:** Differences in dose constraint values between HT and ST techniques. Each column represents a patient subgroup, and the numeric values indicate the differences in mean dose constraint values (Gy or %) between the HT and ST techniques. For OAR, green cells indicate significant negative differences, while red cells indicate significant positive differences. In contrast, for target volumes, green cells indicate significant positive differences, and red cells indicate significant negative differences. Superscript letters: g = green cells, r = red cells.

		(HT R) ‐ (ST R)	(HT L) ‐ (ST L)	(HT R + C) ‐ (ST R + C)	(HT L + C) ‐ (ST L + C)	(HT R + IMN) ‐(ST R + IMN)	(HT L + IMN) ‐(ST L + IMN)
**Ipsilateral Lung**	**Mean Dose (Gy)**	‐2.35 (6.98‐9.33)^g^	‐1.14 (8.07‐9.21)	‐1.60 (13.70‐15.30)	‐1.80 (10.15‐11.95)	‐2.47 (12.12‐14.59) ^g^	‐3.29 (9.43‐12.72) ^g^
	**V**_**5Gy**_ **(%)**	‐7.53 (26.04‐33.57) ^g^	‐2.54 (29.91‐32.45)	‐6.26 (75.14‐81.40)	‐4.63 (59.05‐63.68)	‐4.47 (70.06‐74.53)	‐14.18 (54.76‐68.94) ^g^
	**V**_**20Gy**_ **(%)**	‐5.89 (13.44‐19.33) ^g^	‐3.50 (17.00‐20.50)	‐5.37 (19.40‐24.77)	‐6.16 (15.62‐21.78)	‐6.88 (17.13‐24.01) ^g^	‐8.54 (14.60‐23.14) ^g^
	**V**_**30Gy**_ **(%)**	‐5.45 (8.83‐14.28) ^g^	‐3.86 (11.12‐14.98) ^g^	‐1.66 (12.34‐14.00)	‐2.82 (10.93‐13.75)	‐5.22 (9.63‐14.85) ^g^	‐6.41 (8.80‐15.21) ^g^

**Heart**	**Mean Dose (Gy)**	‐0.10 (0.46‐0.56)	0.11 (1.27‐1.16)	0.21 (2.98‐2.77)	‐0.20 (4.25‐4.45)	0.91 (2.96‐2.05)^r^	‐0.46 (4.19‐4.65) ^g^
	**V**_**20Gy**_ **(%)**	0.00 (0.00‐0.00)	0.24 (0.98‐0.74)	‐0.06 (0.04‐0.10)	‐0.16 (0.57‐0.73)	0.16 (0.23‐0.07)	‐0.41 (0.30‐0.71)
	**V**_**30Gy**_ **(%)**	0.00 (0.00‐0.00)	0.13 (0.45‐0.32)	0.00 (0.00‐0.00)	‐0.06 (0.27‐0.33)	0.01 (0.01‐0.00)	‐0.11 (0.14‐0.25)
	**V**_**35Gy**_ **(%)**	0.00 (0.00‐0.00)	0.06 (0.24‐0.18)	0.00 (0.00‐0.00)	‐0.03 (0.15‐0.18)	0.00 (0.00‐0.00)	‐0.05 (0.09‐0.14)

**CTV Breast or CW**	**Mean Dose (Gy)**	0.70 (48.61‐47.91)	0.78 (47.61‐46.83)	‐0.25 (46.08‐46.33)	‐0.03 (45.82‐45.85)	0.85 (47.00‐46.15)	0.16 (46.28‐46.12)
	**D**_**95%**_ **(Gy)**	1.70 (40.59‐38.89)	2.60 (36.83‐34.23)	‐0.01 (32.16‐32.17)	‐0.20 (29.80‐30.00)	2.36 (33.45‐31.09)	‐2.17 (29.81‐31.98)
	**D**_**90%**_ **(Gy)**	0.98 (44.51‐43.53)	2.35 (42.00‐39.65)	‐1.03 (36.94‐37.97)	‐0.27 (36.08‐36.35)	1.88 (39.29‐37.41)	‐1.18 (36.44‐37.62)
	**V**_**47.5Gy**_ **(%)**	3.81 (76.99‐73.18)	2.76 (69.58‐66.82)	‐4.23 (59.10‐63.33)	‐0.63 (61.90‐62.53)	4.48 (67.72‐63.24)	4.00 (65.84‐61.84)
	**V**_**45Gy**_ **(%)**	2.42 (88.83‐86.41)	3.34 (82.82‐79.48)	‐6.40 (68.70‐75.10)	0.39 (73.47‐73.08)	2.68 (78.67‐75.99)	1.57 (74.80‐73.23)
	**V**_**42.75Gy**_ **(%)**	2.08 (92.66‐90.58)	3.72 (88.62‐84.90)	‐5.90 (75.10‐81.00)	0.30 (78.73‐78.43)	2.56 (83.73‐81.17)	0.84 (79.89‐79.05)
	**V**_**40.5Gy**_ **(%)**	1.80 (94.94‐93.14)	3.35 (91.83‐88.48)	‐3.70 (81.60‐85.30)	0.10 (83.13‐83.03)	2.23 (87.62‐85.39)	0.05 (84.10‐84.05)

**(CTV Breast or CW) - 5mm from skin surface**	**Mean Dose (Gy)**	0.81 (49.65‐48.84) ^g^	0.77 (48.71‐47.94)	0.05 (47.28‐ 7.23)	‐0.03 (47.07‐4710)	1.03 (47.90‐46.87)	0.03 (47.28‐47.25)
	**D**_**95%**_ **(Gy)**	2.15 (46.05‐43.90)	3.07 (41.94‐38.87)	‐1.25 (32.18‐33.43)	‐1.97 (30.08‐32.05)	2.36 (35.74‐33.38)	‐2.58 (30.49‐33.07)
	**D**_**90%**_ **(Gy)**	0.85 (47.06‐46.21)	1.15 (44.77‐43.62)	0.35 (40.98‐40.63)	0.50 (39.50‐39.00)	2.01 (41.93‐39.92)	‐1.32 (38.51‐39.83)
	**V**_**47.5Gy**_ **(%)**	4.64 (86.83‐82.19)	4.04 (80.41‐76.37)	‐0.57 (72.06‐72.63)	2.88 (74.63‐71.75)	5.99 (74.64‐68.65)	3.17 (75.91‐72.74)
	**V**_**45Gy**_ **(%)**	2.85 (97.35‐94.50)	4.37 (92.47‐88.10)	‐4.18 (78.52‐82.70)	3.50 (84.70‐81.20)	5.07 (86.40‐81.33)	0.19 (82.73‐82.54)
	**V**_**42.75Gy**_ **(%)**	2.36 (99.09‐96.73)	4.82 (96.39‐91.57)	‐4.98 (81.72‐86.70)	2.23 (87.43‐85.20)	3.56 (89.53‐85.97)	‐0.39 (85.96‐86.35)
	**V**_**40.5Gy**_ **(%)**	2.01 (99.50‐97.49)	4.06 (97.51‐93.45)	‐3.24 (86.36‐89.60)	1.02 (89.42‐88.40)	2.82 (91.82‐89.00)	‐0.66 (88.65‐89.31)

**Supraclavicular**	**Mean Dose (Gy)**	‐2.04 (40.30‐42.34)	‐1.62 (41.83‐43.45)	0.82 (43.92‐43.10)	‐0.10 (43.10‐43.20)	‐1.88 (40.83‐42.71) ^r^	‐2.44 (40.91‐43.35) ^r^
	**D**_**95%**_ **(Gy)**	‐1.36 (32.26‐33.62)	‐0.26 (34.76‐35.02)	1.26 (35.96‐34.70)	0.23 (35.88‐35.65)	‐0.49 (33.85‐34.34)	‐1.45 (33.81‐35.26)
	**D**_**90%**_ **(Gy)**	‐1.34 (35.39‐36.73)	‐0.67 (37.18‐37.85)	1.20 (39.24‐38.03)	0.70 (39.00‐38.30)	‐0.76 (36.78‐37.54)	‐1.80 (36.78‐38.58)
	**V**_**47.5Gy**_ **(%)**	‐3.19 (1.35‐4.54)	‐4.65 (0.18‐4.83) ^r^	2.71 (8.78‐6.07)	‐1.83 (2.10‐3.93)	‐2.62 (0.70‐3.32)	‐9.65 (0.06‐9.71)
	**V**_**45Gy**_ **(%)**	‐19.90 (11.93‐31.83)	‐19.98 (21.30‐41.28) ^r^	14.37 (49.30‐34.93)	‐4.72 (33.83‐38.55)	‐23.40 (11.18‐34.58) ^r^	‐24.62 (14.63‐39.25) ^r^
	**V**_**42.75Gy**_ **(%)**	‐27.07 (28.50‐55.57)	‐15.42 (50.07‐65.49)	9.88 (73.98‐64.10)	‐5.17 (62.93‐68.10)	‐28.34 (33.43‐61.77) ^r^	‐28.80 (35.16‐63.96) ^r^
	**V**_**40.5Gy**_ **(%)**	‐21.89 (52.30‐74.19)	‐5.91 (72.37‐78.28)	3.67 (86.20‐82.53)	0.05 (82.38‐82.33	‐18.88 (59.65‐78.53) ^r^	‐22.39 (58.91‐81.30) ^r^

**Axillary Level I**	**Mean Dose (Gy)**	5.46 (42.39‐36.93) ^g^	5.76 (42.18‐36.42) ^g^	1.35 (44.58‐43.23)	2.22 (46.15‐43.93)	5.43 (45.83‐40.40) ^g^	4.70 (47.25‐42.55) ^g^
	**D**_**95%**_ **(Gy)**	6.62 (23.31‐16.69)	11.60 (24.78‐13.18) ^g^	‐2.23 (31.60‐33.83)	1.97 (35.27‐33.30)	9.53 (34.48‐24.95) ^g^	8.33 (39.18‐30.85) ^g^
	**D**_**90%**_ **(Gy)**	6.99 (29.23‐22.24)	9.83 (30.89‐21.06) ^g^	‐0.85 (36.38‐37.23)	3.85 (40.60‐36.75)	8.72 (38.44‐29.72) ^g^	7.78 (42.23‐34.45) ^g^
	**V**_**47.5Gy**_ **(%)**	27.86 (34.58‐6.72) ^g^	15.90 (26.00‐10.10) ^g^	26.97 (43.10‐16.13) ^g^	16.09 (49.72‐33.63)	37.76 (50.93‐13.17) ^g^	34.77 (63.64‐28.87) ^g^
	**V**_**45Gy**_ **(%)**	35.88 (56.39‐20.51) ^g^	29.85 (51.29‐21.44) ^g^	14.15 (58.18‐44.03)	20.37 (73.95‐53.58)	38.53 (70.66‐32.13) ^g^	32.16 (79.21‐47.05) ^g^
	**V**_**42.75Gy**_ **(%)**	37.43 (71.09‐33.66) ^g^	35.77 (66.65‐30.88) ^g^	5.91 (71.04‐65.13)	18.40 (82.90‐64.50) ^g^	32.74 (78.77‐46.03) ^g^	30.81 (85.01‐54.20) ^g^
	**V**_**40.5Gy**_ **(%)**	31.05 (77.23‐46.18) ^g^	33.37 (75.43‐42.06) ^g^	1.17 (81.24‐80.07)	12.62 (88.70‐76.08)	24.68 (84.54‐59.86) ^g^	25.16 (91.03‐65.87) ^g^

**Axillary Level II**	**Mean Dose (Gy)**	0.12 (40.04‐39.92)	1.93 (42.03‐40.10	1.73 (43.40‐41.67)	0.67 (41.85‐41.18)	‐0.51 (40.82‐41.33)	1.31 (41.91‐40.60)
	**D**_**95%**_ **(Gy)**	‐8.42 (25.64‐34.06)	‐1.52 (32.10‐33.62)	4.22 (37.42‐33.20)	‐1.98 (31.50‐33.48)	1.35 (32.06‐30.71)	‐3.68 (30.65‐34.33)
	**D**_**90%**_ **(Gy)**	‐6.13 (29.65‐35.78)	‐1.25 (34.45‐35.70)	2.36 (39.26‐36.90)	‐2.15 (34.38‐36.53)	0.02 (34.25‐34.23)	‐2.46 (33.69‐36.15)
	**V**_**47.5Gy**_ **(%)**	20.42 (21.04‐0.62) ^g^	17.74 (19.77‐2.03) ^g^	11.25 (17.28‐6.03)	17.05 (22.50‐5.45)	11.69 (15.27‐3.58) ^g^	20.80 (22.29‐1.49) ^g^
	**V**_**45Gy**_ **(%)**	22.95 (30.79‐7.84) ^g^	23.64 (31.70‐8.06) ^g^	21.12 (44.72‐23.60)	13.07 (33.85‐20.78)	8.21 (22.67‐14.46)	23.76 (33.64‐9.88) ^g^
	**V**_**42.75Gy**_ **(%)**	16.10 (40.71‐24.61)	18.47 (44.10‐25.63) ^g^	20.97 (63.44‐42.47)	10.45 (50.95‐40.50)	‐5.59 (33.23‐38.82)	19.25 (47.66‐28.41)
	**V**_**40.5Gy**_ **(%)**	3.60 (53.54‐49.94)	9.26 (61.04‐51.78)	11.63 (80.30‐68.67)	0.80 (63.50‐62.70)	‐14.68 (49.62‐64.30)	3.20 (61.06‐57.86)

**Axillary Level III**	**Mean Dose (Gy)**	‐2.19 (39.50‐41.69)	‐1.18 (40.99‐42.17)	1.11 (43.94‐42.83)	‐1.03 (40.82‐41.85)	‐2.29 (41.13‐43.42) ^r^	‐2.28 (39.74‐42.02)
	**D**_**95%**_ **(Gy)**	‐5.80 (32.58‐38.38) ^r^	‐2.72 (36.66‐39.38) ^r^	1.73 (41.70‐39.97)	‐2.55 (35.53‐38.08)	4.73 (37.62‐32.89) ^g^	‐2.68 (35.08‐37.76)
	**D**_**90%**_ **(Gy)**	‐5.26 (34.25‐39.51) ^r^	‐2.34 (37.64‐39.98) ^r^	1.59 (42.22‐40.63)	‐1.55 (37.08‐38.63)	1.52 (38.38‐36.86)	‐2.59 (36.36‐38.95)
	**V**_**47.5Gy**_ **(%)**	5.87 (5.94‐0.07)	1.92 (2.22‐0.30)	3.88 (3.88‐0.00)	‐0.38 (0.12‐0.50)	‐20.54 (0.10‐20.64) ^r^	‐2.12 (0.25‐2.37)
	**V**_**45Gy**_ **(%)**	3.94 (13.58‐9.64)	1.26 (8.87‐7.61)	23.63 (47.36‐23.73)	‐2.63 (8.22‐10.85)	‐37.5 (2.61‐40.11) ^r^	‐7.41 (6.61‐14.02)
	**V**_**42.75Gy**_ **(%)**	‐18.43 (19.30‐37.73)	‐11.04 (29.75‐40.79)	12.87 (66.14‐53.27)	‐16.83 (34.55‐51.38)	‐45.97 (18.68‐64.65) ^r^	‐27.63 (18.79‐46.42)
	**V**_**40.5Gy**_ **(%)**	‐33.61 (34.31‐67.92) ^r^	‐19.97 (59.75‐79.72)	3.48 (84.58‐81.10)	‐17.40 (61.13‐78.53)	‐25.47 (56.66‐82.13) ^r^	‐36.71 (41.21‐77.92) ^r^

**IMN**	**Mean Dose (Gy)**	‐1.01 (11.45‐12.46)	3.89 (15.79‐11.90)	‐8.82 (29.08‐37.90) ^r^	‐10.38 (19.27‐29.65) ^r^	‐0.21 (33.20‐33.41)	‐0.20 (28.81‐29.01)
	**D**_**95%**_ **(Gy)**	‐1.06 (2.33‐3.39)	3.28 (7.68‐4.40)	‐5.54 (21.16‐26.70)	‐2.31 (10.72‐13.03)	‐3.53 (20.27‐23.80)	3.47 (16.56‐13.09) ^g^
	**D**_**90%**_ **(Gy)**	‐2.01 (2.81‐4.82)	2.64 (8.59‐5.95)	‐6.89 (22.98‐29.87)	‐6.13 (11.65‐17.78)	‐1.22 (23.70‐24.92)	3.03 (19.26‐16.23)
	**V**_**47.5Gy**_ **(%)**	‐1.21 (0.00‐1.21)	1.64 (7.54‐5.90)	0.00 (0.00‐0.00)	0.00 (0.00‐0.00)	0.00 (0.00‐0.00)	0.00 (0.00‐0.00)
	**V**_**45Gy**_ **(%)**	‐2.50 (0.08‐2.58)	2.03 (9.35‐7.32)	‐4.14 (0.26‐4.40)	‐0.18 (0.00‐0.18)	0.35 (1.18‐0.83)	‐1.89 (0.35‐2.24)
	**V**_**42.75Gy**_ **(%)**	‐3.68 (0.26‐3.94)	2.94 (11.26‐8.32)	‐12.40 (4.80‐17.20)	‐3.28 (0.00‐3.28)	1.61 (9.25‐7.64)	‐6.43 (2.11‐8.54)
	**V**_**40.5Gy**_ **(%)**	‐4.60 (0.86‐5.46)	3.52 (13.08‐9.56)	‐28.41 (11.46‐39.87)	‐6.68 (0.00‐6.68)	3.33 (20.51‐17.18)	‐11.01 (5.54‐16.55)


Figure 3Boxplots illustrating various dose constraint values for each organ at risk (OAR) and target volume across all subgroups in the two techniques: ST and HT. (a) Ipsilateral lung, (b) heart, (c) CTV breast or chest wall (CW), (d) CTV breast or CW excluding 5 mm from the skin surface, (e) supraclavicular region, (f) axillary level I, (g) axillary level II, (h) axillary level III, and (i) internal mammary nodes (IMN). The thick horizontal line within each box represents the median (50th percentile) of the dose constraint values for the respective technique subgroup. The lower and upper edges of the box indicate the first quartile (Q1, 25th percentile) and third quartile (Q3, 75th percentile), respectively. Together, the box represents the interquartile range (IQR), covering the middle 50% of the data. The whiskers (vertical lines extending from the box) represent the range of data within Q1 − 1.5 × IQR and Q3 + 1.5 × IQR. Data points outside this range are plotted as individual dots or circles and are considered outliers.

**Figure:**
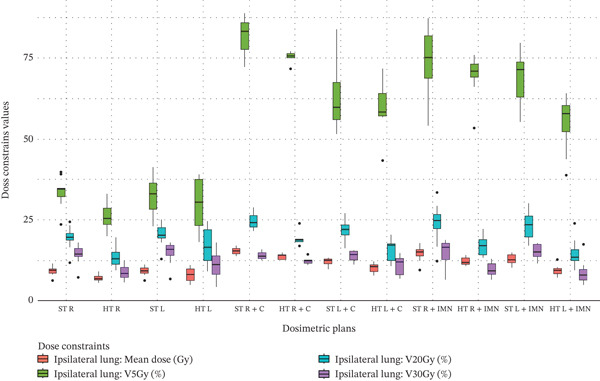
(a)

**Figure:**
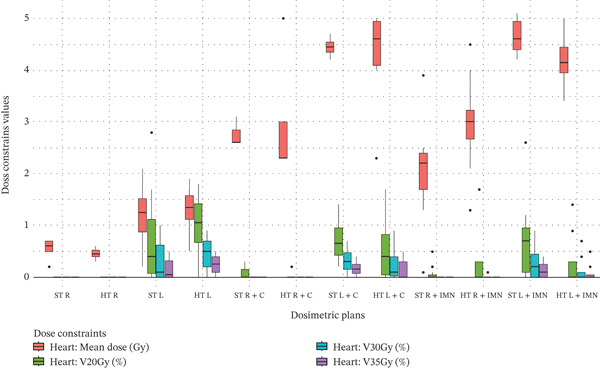
(b)

**Figure:**
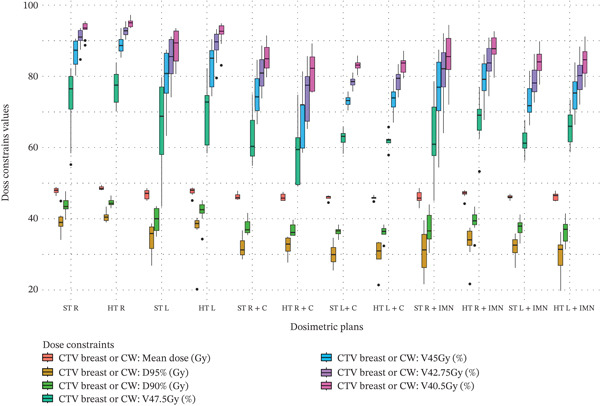
(c)

**Figure:**
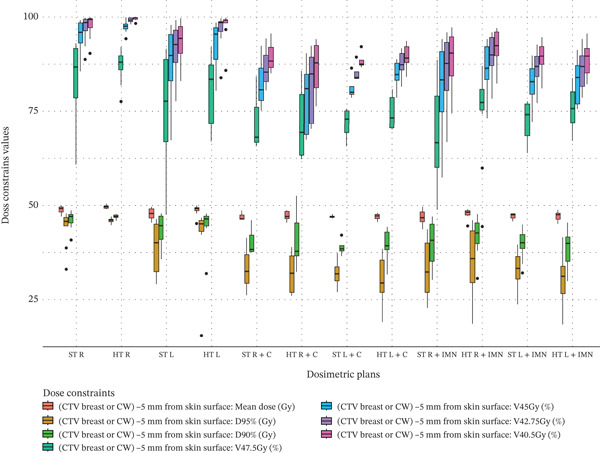
(d)

**Figure:**
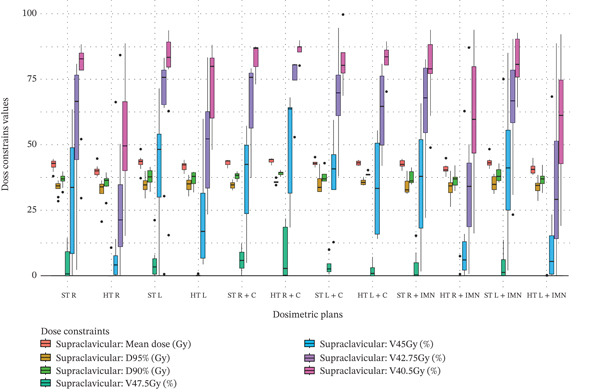
(e)

**Figure:**
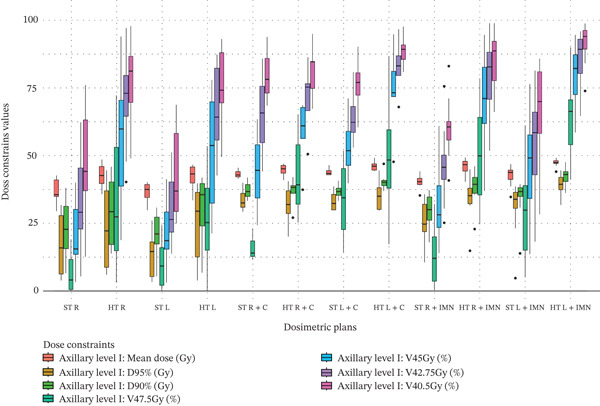
(f)

**Figure:**
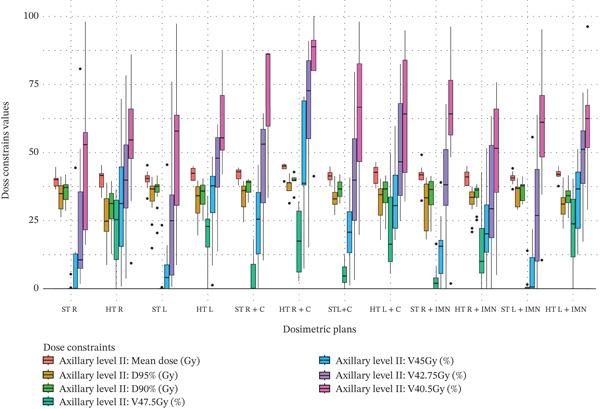
(g)

**Figure:**
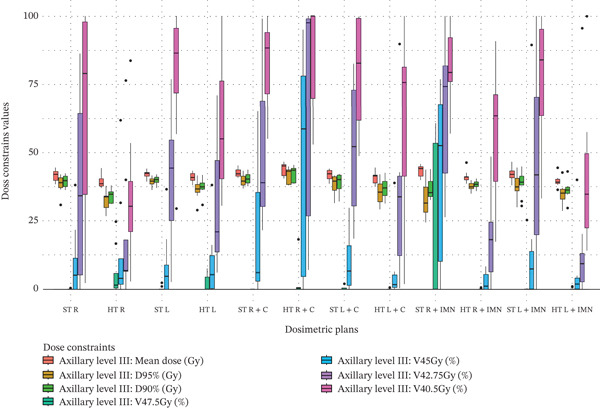
(h)

**Figure:**
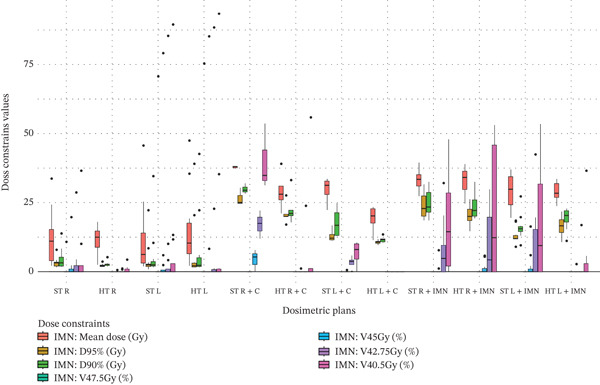
(i)

### 3.3. Heart

Regarding the heart, significant differences between the HT and ST techniques were detected only for the mean dose constraint when the IMN was included using a dedicated field, as shown in Table 3. The distribution of all data is illustrated in Figure 3b. When the IMN field was included, a significant improvement in heart mean dose by 0.46 Gy was observed for the left side, indicated by the green cell (Table 3). However, for the right side, a significant deterioration was observed, with the mean dose increasing by 0.91 Gy, as indicated by the red cell.

### 3.4. CTV Breast or CW

For the CTV of the breast or CW, there were no significant differences in dose constraint values between the HT and ST techniques (no green or red cells in Table 3). The data distribution is shown in Figure 3c. However, when 5 mm was excluded from the skin surface, represented by the “(CTV Breast or CW) ‐ 5 mm from skin surface” section of Table 3, a significant coverage improvement of 0.81 Gy was observed only for the mean dose of right‐sided breast or CW plans when the IMN or C were not included. The corresponding data distribution is shown in Figure 3d.

### 3.5. Supraclavicular

For the supraclavicular region, clear and significant coverage deteriorations (red cells) were observed, except when the C field was used or for the right side when neither IMN nor C fields were included, as shown in Table 3. The data distribution is visualized in Figure 3e. For the left side without inclusion of IMN or C fields, dose constraint values were significantly reduced by 4.65% and 19.98% for V_47.5Gy_ and V_45Gy_, respectively. When the IMN was included using a dedicated field, dose constraint values were significantly reduced as follows: for the right side, by 1.88 Gy, 23.40%, 28.34%, and 18.88% for mean dose, V_45Gy_, V_42.75Gy_, and V_40.5Gy_, respectively; and for the left side, by 2.44 Gy, 24.62%, 28.80%, and 22.39% for the same respective metrics.

### 3.6. Axillary Level I

Clear and significant improvements in axillary level I coverage were observed across all columns of values in Table 3. The data distribution is shown in Figure 3f. When IMN or C fields were not included, coverage improvements were as follows: for the right side, 5.46 Gy, 27.86%, 35.88%, 37.43%, and 31.05% for mean dose, V_47.5Gy_, V_45Gy_, V_42.75Gy_, and V_40.5Gy_, respectively; and for the left side, 5.76 Gy, 11.60 Gy, 9.83 Gy, 15.90%, 29.85%, 35.77%, and 33.37% for mean dose, D_95%_, D_90%_, V_47.5Gy_, V_45Gy_, V_42.75Gy_, and V_40.5Gy_, respectively. When the C field was included, coverage improvement was observed only for V_47.5Gy_ on the right side (26.97%) and for V_42.75Gy_ on the left side (18.40%). When the IMN field was included using a dedicated field, improvements were as follows: for the right side, 5.43 Gy, 9.53 Gy, 8.72 Gy, 37.76%, 38.53%, 32.74%, and 24.68% for mean dose, D_95%_, D_90%_, V_47.5Gy_, V_45Gy_, V_42.75Gy_, and V_40.5Gy_, respectively; and for the left side, 4.70 Gy, 8.33 Gy, 7.78 Gy, 34.77%, 32.16%, 30.81%, and 25.16% for the same respective metrics.

### 3.7. Axillary Level II

Regarding axillary level II, significant coverage improvements were observed across the columns of values in Table 3, excluding those corresponding to the C field. The data distribution for axillary level II is illustrated in Figure 3g. For breast or CW plans that did not include the IMN or C fields, axillary level II coverage increased as follows: for the right side, by 20.42% for V_47.5Gy_ and 22.95% for V_45Gy_; for the left side, by 17.74% for V_47.5Gy_, 23.64% for V_45Gy_, and 18.47% for V_42.75Gy_. Furthermore, when the IMN field was included using a dedicated field, significant improvements were observed as follows: for the right side, by 11.69% for V_47.5Gy_; and for the left side, by 20.80% for V_47.5Gy_ and 23.76% for V_45Gy_.

### 3.8. Axillary Level III

For axillary level III, significant coverage deterioration was observed across the columns of values in Table 3, excluding those corresponding to the C field. However, a significant coverage improvement of 4.73 Gy was observed only for D_95%_ of right‐side plans when the IMN was included using a dedicated field. The data distribution for axillary level III is illustrated in Figure 3h. For breast or CW plans that did not include the IMN or C fields, axillary level III coverage decreased as follows: for the right side, by 5.8 Gy for D_95%_, 5.26 Gy for D_90%_, and 33.61% for V_40.5Gy_; for the left side, by 2.72 Gy for D_95%_ and 2.34 Gy for D_90%_. Furthermore, when the IMN field was included using a dedicated field, significant deteriorations were observed as follows: for the right side, by 2.29 Gy for mean dose, 20.54% for V_47.5Gy_, 37.5% for V_45Gy_, 45.97% for V_42.75Gy_, and 25.47% for V_40.5Gy_; and for the left side, by 36.71% for V_40.5Gy_.

### 3.9. IMN

Significant IMN coverage deterioration was observed when the C field was used without the intention to include the IMN in the treatment. This is indicated by the red cells in the IMN section of Table 3. The observed decreases in IMN coverage were as follows: a reduction of 8.82 Gy in mean dose for the right side, and 10.38 Gy for the left side. However, when the IMN was intentionally targeted, a significant improvement in coverage was observed, with an increase of 3.47 Gy in D_95%_ for the left side. The full visual distribution of IMN dose data is illustrated in Figure 3i.

## 4. Discussion

In breast radiotherapy, the choice of treatment technique should be personalized based on the individual patient’s anatomy [17, 21]. In the present study, among 43 patients requiring IMN irradiation, only one case could be treated with the WT technique according to the guidelines, achieving the most favorable results. This highlights that newer techniques are not always better; rather, the most appropriate approach is the one that best suits the clinical context. Additionally, when OAR dose constraints limit target coverage optimization, for example, when the ipsilateral lung irradiation is restricted to 2 cm by the tangential fields to stay within lung dose constraints, an optimized 3DCRT approach that reduces the high dose to the lung can allow for the inclusion of more than 2 cm of lung. As a result, and in addition to using breathing‐adapted techniques such as DIBH or CPAP, more patients than initially anticipated could potentially be treated with the WT technique, thereby improving target coverage while still respecting OAR dose constraints.

When comparing the present study to the work of Fontanilla et al. [18], who used a technique similar to the ST approach employed here, and like the present study without referencing RTGO contours, which were contoured only after dosimetry for evaluation purposes, some differences are evident. Notably, in their study, the IMN field was treated using electrons alone, rather than a mixed electron‐photon technique as used in the current study. Furthermore, the prescription dose to the lymph nodes was 50 Gy, the same as for the breast or CW. In Fontanilla et al.’s study, for breast or CW the V_45Gy_ was 74%, which have comparable findings in the present study (74.61% for ST and 76.74% for HT). However, their reported ipsilateral lung V_20Gy_ values were higher: 28% for left‐sided and 34% for right‐sided treatments. In contrast, the present study reported 23.14% for ST and 14.60% for HT on the left side, and 24.01% for ST and 17.13% for HT on the right side. The higher OARs dose values in the Fontanilla et al. study are likely attributable to the higher prescribed dose to the lymph nodes. In addition, when comparing the present results to those reported by Goyal et al. [19], better target coverage was observed in their study, but at the expense of higher doses to OARs. For left‐sided treatments, Goyal et al. reported a mean heart dose of 7.98 Gy, compared with 4.65 Gy for ST and 4.19 Gy for HT in the present study. The heart V_30Gy_ was 5.75%, whereas in the present study, it was 0.25% for ST and 0.14% for HT. The left lung mean dose in their study was 15.95 Gy, while in the present study, it was 12.72 Gy for ST and 9.43 Gy for HT. Similarly, their left lung V_20Gy_ was 31.64%, compared with 23.14% for ST and 14.60% for HT in the current study. Furthermore, in the study by Jalbout et al. [17], the CT acquisition method, whether free‐breathing or breathing‐adapted techniques such as DIBH or CPAP was not specified, an important consideration, as such breathing‐adapted techniques can reduce OAR doses [7]. Additionally, the IMN field used electrons only, without photon mixing, and the global maximum dose was not constrained to 110% of the prescribed dose as in the present study (55 Gy); instead, it reached 70.9 Gy, which represent 141.8% of the prescribed 50 Gy. These were major factors contributing to their superior targets coverage compared with the current study findings. Jalbout et al. also divided patients into two groups based on whether the left anterior descending artery (LAD) was included in the WT fields. For the group with the lowest OAR exposure, the mean heart dose was 2.1 Gy (compared with 4.65 Gy for ST and 4.19 Gy for HT in our study), likely due to the use of full‐electron IMN fields. However, their left lung V_20Gy_ was 22.0% (vs. 23.14% for ST and 14.60% for HT in our study), and the mean lung dose was 11.5 Gy (vs. 12.72 Gy for ST and 9.43 Gy for HT). Moreover, when the WT technique was used in Jalbout et al.’s study, even better target coverage was achieved for the group with the lowest doses to OARs, with a mean heart dose of only 1.5 Gy (compared with 4.65 Gy for ST and 4.19 Gy for HT). However, other OAR constraints were still better in the current study, including a left lung V_20Gy_ of 20.5% (vs. 23.14% for ST and 14.60% for HT) and a mean lung dose of 10.7 Gy (vs. 12.72 Gy for ST and 9.43 Gy for HT). Additionally, the global maximum dose of WT group in their study was 56.8 Gy (113.6% of 50 Gy), which remained acceptable but was slightly higher than the 110% limit used in the present study. Consequently, to further improve coverage outcomes, future studies may consider adjusting the HT technique by using a fully electron‐based IMN (or C) field instead of the mixed electron–photon approach, and by relaxing the maximum dose constraint beyond the current 110% of the prescribed dose.

A global overview of the most prominent and statistically significant results comparing the ST and HT techniques (Table 3) indicates that, compared with ST, HT improves ipsilateral lung dose and axillary levels I and II coverage (cells highlighted in green). However, HT results in reduced coverage of the supraclavicular region and axillary level III (cells highlighted in red). To investigate this decline in coverage, a schematic representation that reveals an exaggerated tilt of the supraclavicular beam associated with a high breast board angle is illustrated in Figure 4a. The original beam is shown in green, while the tilted version appears in red. As shown in Figure 4b, tilting the beam reduces its superior–inferior dimension in order to align with the superior and inferior borders of the clavicle, as described in the Methods and Materials section. Consequently, the supraclavicular region (cyan in Figure 4) and axillary level III (blue) are not fully covered by the tilted beam. Therefore, to improve coverage, future studies should consider adjusting the beam orientation according to the targets contours rather than the borders of the clavicle. This approach was demonstrated by Fontanilla et al. [18], who compared two sets of dosimetry plans: one generated without RTOG contour guidance and the other created with RTOG contours available during planning. Their findings showed that incorporating RTOG contours during planning significantly improved target coverage. On the other hand, as mentioned in the Introduction, Figure 4 clearly demonstrates that the tilted field irradiates less lung tissue than the original field. Additionally, the figure shows that the original supraclavicular field is perpendicular to the skin surface; however, when tilted, it is no longer perpendicular, which increases the depth that must be traversed to reach the supraclavicular and axillary level II targets [22]. Consequently, limiting the maximum dose in the supraclavicular field to 108% of the prescribed 45 Gy may be an additional factor restricting adequate coverage of deeper targets. Therefore, in future studies, this maximum dose constraint should be reconsidered and possibly relaxed, similar to the global maximum dose discussed earlier.

Figure 4Schematic representation of an exaggeratedly tilted supraclavicular beam associated with a high breast board angle and its effect on target coverage. (a) The original supraclavicular beam is shown in green; the tilted supraclavicular beam in red; the supraclavicular region in cyan; axillary level III in blue; and, within the patient outline, the ipsilateral lung and clavicle in black. (b) Zoomed‐in view showing a clear reduction in the superior‐inferior beam dimension when tilted, resulting in inadequate coverage of the supraclavicular region and axillary level III.

**Figure:**
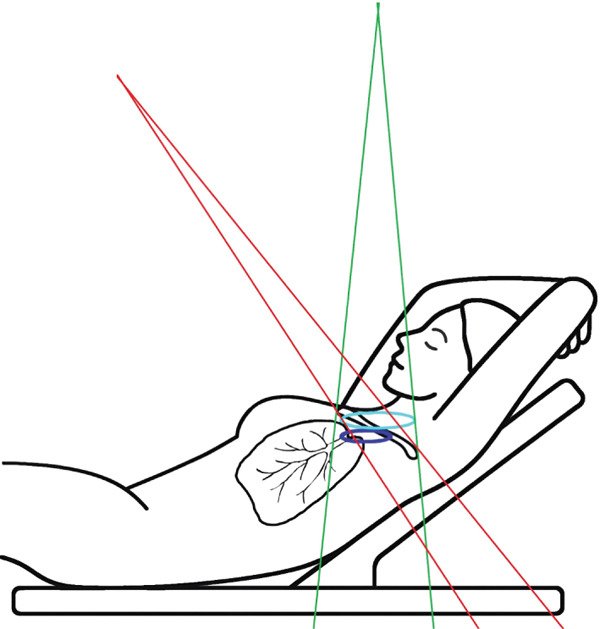
(a)

**Figure:**
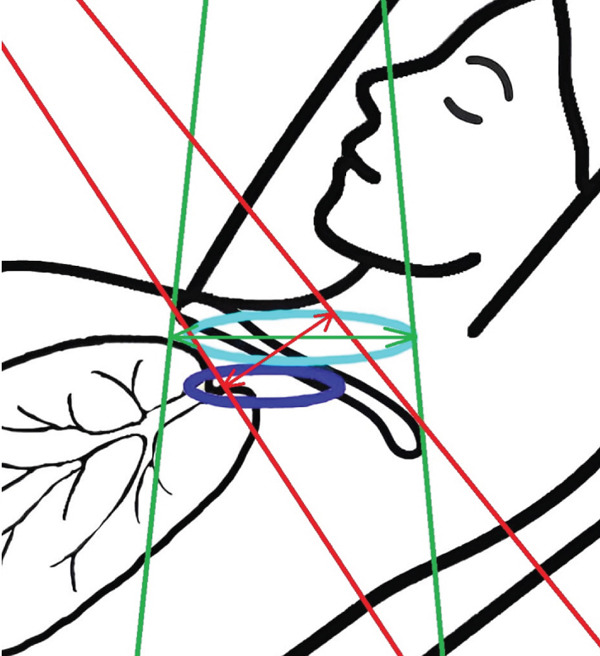
(b)

Other factors may be considered in future studies. For example, to facilitate patient positioning during treatment and avoid overlaps associated with multi‐isocentric techniques, it is recommended to use a monoisocentric approach with HT. However, as highlighted in the present study, during the dosimetry planning process, the tangential fields should initially be positioned as superiorly as possible to create an intentional internal overlap with the supraclavicular field. This overlap can then be resolved in a second step by applying the FIF technique to the supraclavicular field. Additionally, including a larger number of patients may yield more statistically significant results. Although the current study included 100 patients, they were divided into different groups, which reduced the sample size per group and limited the statistical power. Finally, when the missing factors identified in this study are incorporated into the current HT 3DCRT technique, a future study should compare this approach with advanced techniques such as VMAT. This comparison should involve a protocol in which dosimetry is performed for each patient using both techniques, with dosimetric parameters recorded for each, along with the technique ultimately selected by the radiation oncologist for treatment.

## 5. Conclusions

The HT 3DCRT technique shows promising results. This approach involves tilting the supraclavicular beam to minimize lung dose, using a superiorly positioned HTF to improve coverage of axillary levels I and II, and intentionally creating an internal overlap between the supraclavicular and tangential fields, subsequently resolved using the FIF technique, primarily applied to the supraclavicular field, which contributes most to the ipsilateral lung dose in this region. However, several additional considerations must be addressed before recommending the HT technique for routine clinical use and presenting its dosimetry to radiation oncologists:

- Targets and OARs contouring should be completed prior to dosimetric planning to enable proper field adjustment.
- Relaxing the 2 cm constraint on ipsilateral lung irradiation by the tangential field when the IMN is included, thereby increase the probability of allowing the use of the WT technique, which is preferred over the IMN dedicated field technique.
- To optimize coverage of the supraclavicular region, axillary level III, and the IMN, the maximum dose constraint should be relaxed for both the supraclavicular field and any IMN dedicated field, if used.
- When the IMN dedicated technique or C field are selected, a fully electron‐based field should be used instead of a mixed electron‐photon approach.

## Funding

No funding was received for this research.

## Conflicts of Interest

The authors declare no conflicts of interest.

## Supporting information


**Supporting Information 1** Additional supporting information can be found online in the Supporting Information section. Table S1. The dosimetric data for all 100 patients included in the study, providing detailed dose parameters for target volumes and OARs.

## Data Availability

Data available in article supporting information.

## References

[1] Bray F. , Laversanne M. , Sung H. , Ferlay J. , Siegel R. L. , Soerjomataram I. , and Jemal A. , Global Cancer Statistics 2022: GLOBOCAN Estimates of Incidence and Mortality Worldwide for 36 Cancers in 185 Countries, CA: A Cancer Journal for Clinicians. (2024) 74, no. 3, 229–263, 10.3322/caac.21834, 38572751. 38572751

[2] Miller K. D. , Nogueira L. , Devasia T. , Mariotto A. B. , Yabroff K. R. , Jemal A. , Kramer J. , and Siegel R. L. , Cancer Treatment and Survivorship Statistics, 2022, CA: A Cancer Journal for Clinicians. (2022) 72, no. 5, 409–436, 10.3322/caac.21731, 35736631. 35736631

[3] Taylor C. , Dodwell D. , McGale P. , Hills R. K. , Berry R. , Bradley R. , Braybrooke J. , Clarke M. , Gray R. , Holt F. , Liu Z. , Pan H. , Peto R. , Straiton E. , Coles C. , Duane F. , Hennequin C. , Jones G. , Kühn T. , Oliveros S. , Overgaard J. , Pritchard K. I. , Suh C. O. , Beake G. , Boddington C. , Davies C. , Davies L. , Evans V. , Gay J. , Gettins L. , Godwin J. , James S. , Kerr A. , Liu H. , MacKinnon E. , Mannu G. , McHugh T. , Morris P. , Nakahara M. , Read S. , Taylor H. , Ferguson J. , Scheurlen H. , Zurrida S. , Galimberti V. , Ingle J. , Valagussa P. , Veronesi U. , Anderson S. , Tang G. , Fisher B. , Fossa S. , Valborg Reinertsen K. , Host H. , Muss H. , Holli K. , Albain K. , Arriagada R. , Bartlett J. , Bergsten-Nordström E. , Bliss J. , Brain E. , Carey L. , Coleman R. , Cuzick J. , Davidson N. , del Mastro L. , di Leo A. , Dignam J. , Dowsett M. , Ejlertsen B. , Francis P. , García-Sáenz J. A. , Gelber R. , Gnant M. , Goetz M. , Goodwin P. , Halpin-Murphy P. , Hayes D. , Hill C. , Jagsi R. , Janni W. , Loibl S. , Mamounas E. , Martín M. , McIntosh S. , Mukai H. , Nekljudova V. , Norton L. , Ohashi Y. , Piccart M. , Pierce L. , Raina V. , Rea D. , Regan M. , Robertson J. , Rutgers E. , Salgado R. , Slamon D. , Spanic T. , Sparano J. , Steger G. , Toi M. , Tutt A. , Viale G. , Wang X. , Wilcken N. , Wolmark N. , Yu K. D. , Cameron D. , Bergh J. , Swain S. , Whelan T. , and Poortmans P. , Radiotherapy to Regional Nodes in Early Breast Cancer: An Individual Patient Data Meta-Analysis of 14 324 Women in 16 Trials, Lancet. (2023) 402, no. 10416, 1991–2003, 10.1016/S0140-6736(23)01082-6. 37931633

[4] Luo J. , Jin K. , Chen X. , Wang X. , Yang Z. , Zhang L. , Mei X. , Ma J. , Zhang X. , Zhou Z. , Wang X. , Jiang Y. , Shao Z. , Zhang Z. , Guo X. , and Yu X. , Internal Mammary Node Irradiation (IMNI) Improves Survival Outcome for Patients With Clinical Stage II–III Breast Cancer After Preoperative Systemic Therapy, International Journal of Radiation Oncology • Biology • Physics. (2019) 103, no. 4, 895–904, 10.1016/j.ijrobp.2018.11.003, 2-s2.0-85059945977. 30439485

[5] Aznar M. C. , Duane F. K. , Darby S. C. , Wang Z. , and Taylor C. W. , Exposure of the Lungs in Breast Cancer Radiotherapy: A Systematic Review of Lung Doses Published 2010–2015, Radiotherapy and Oncology. (2018) 126, no. 1, 148–154, 10.1016/j.radonc.2017.11.022, 2-s2.0-85049410319, 29246585. 29246585 PMC5807032

[6] Verginadis I. I. , Citrin D. E. , Ky B. , Feigenberg S. J. , Georgakilas A. G. , Hill-Kayser C. E. , Koumenis C. , Maity A. , Bradley J. D. , and Lin A. , Radiotherapy Toxicities: Mechanisms, Management, and Future Directions, Lancet. (2025) 405, no. 10475, 338–352, 10.1016/S0140-6736(24)02319-5, 39827884. 39827884 PMC12758832

[7] Choi M. S. , Park R. H. , Lee J. , Cho Y. , Chang J. S. , Kim J. , and Kim J. S. , Dosimetric Comparison of CPAP and DIBH for Left-Sided Breast Cancer Radiation Therapy, Advances in Radiation Oncology. (2024) 9, no. 6, 101478, 10.1016/j.adro.2024.101478, 38681894. 38681894 PMC11043855

[8] Balaji K. , Subramanian B. , Yadav P. , Radha C. A. , and Ramasubramanian V. , Radiation Therapy for Breast Cancer: Literature Review, Medical Dosimetry. (2016) 41, no. 3, 253–257, 10.1016/j.meddos.2016.06.005, 2-s2.0-84990866800. 27545009

[9] Abo-Madyan Y. , Aziz M. H. , Aly M. M. , Schneider F. , Sperk E. , Clausen S. , Giordano F. A. , Herskind C. , Steil V. , Wenz F. , and Glatting G. , Second Cancer Risk After 3D-CRT, IMRT and VMAT for Breast Cancer, Radiotherapy and Oncology. (2014) 110, no. 3, 471–476, 10.1016/j.radonc.2013.12.002, 2-s2.0-84899507858, 24444525. 24444525

[10] Wang J. Z. , Li X. A. , D′Souza D. , and W, Stewart RD. , Impact of Prolonged Fraction Delivery Times on Tumor Control: A Note of Caution for Intensity-Modulated Radiation Therapy (IMRT), Physics. (2003) 57, no. 2, 543–552, 10.1016/S0360-3016(03)00499-1, 2-s2.0-0041381126. 12957268

[11] Rossi M. , Boman E. , and Kapanen M. , Optimal Selection of Optimization Bolus Thickness in Planning of VMAT Breast Radiotherapy Treatments, Medical Dosimetry. (2019) 44, no. 3, 266–273, 10.1016/j.meddos.2018.10.001, 2-s2.0-85055649788, 30389413. 30389413

[12] Alço G. , Iğdem S. I. , Ercan T. , Dincer M. , Şentürk R. , Atilla S. , Oral Zengin F. , and Okkan S. , Coverage of Axillary Lymph Nodes With High Tangential Fields in Breast Radiotherapy, British Journal of Radiology. (2010) 83, no. 996, 1072–1076, 10.1259/bjr/25788274, 2-s2.0-78649834547, 21088091. 21088091 PMC3473605

[13] Jung J. , Kong M. , Kim S. S. , and Yoon W. S. , Coverage of Axillary Lymph Nodes With Tangential Breast Irradiation in Korea: A Multi-Institutional Comparison Study, International Journal of Breast Cancer. (2016) 2016, no. 1, 8576357, 10.1155/2016/8576357, 2-s2.0-85006186483. 27525123 PMC4972917

[14] Jacobson G. , Bunda-Randall N. , Wen S. , and Miller M. , Axillary Lymph Node Coverage With 3-Dimensional Tangential Field Irradiation and Correlation With Heart and Lung Dose, Advances in Radiation Oncology. (2017) 2, no. 4, 630–635, 10.1016/j.adro.2017.07.005, 2-s2.0-85028654459, 29204531. 29204531 PMC5707414

[15] Yang B. , Dong Z. , Lin M. H. , and Ma C. M. , A New Method to Deliver Supraclavicular Radiation in Breast Radiotherapy for Lung Sparing, Journal of Applied Clinical Medical Physics. (2011) 12, no. 3, 169–177, 10.1120/jacmp.v12i3.3374, 2-s2.0-80052183891, 21844847. PMC5718654 21844847

[16] Arbab M. , Frame R. , Alluri P. , Parsons D. , Lin M. H. , Cleaton J. , and Rahimi A. , Master Breast Radiation Planning: Simple Guide for Radiation Oncology Residents, Advances in Radiation Oncology. (2024) 9, no. 6, 101476, 10.1016/j.adro.2024.101476, 38690296. 38690296 PMC11059315

[17] Jalbout W. , Youssef B. , and Chahrour Z. , Wide Tangent Photon Field Versus Electron Field in the Treatment of Internal Mammary Lymph Nodes in Patients With Left Breast Cancer: A Decision-Making Flowchart, Advances in Radiation Oncology. (2023) 8, no. 6, 101282, 10.1016/j.adro.2023.101282, 37457821. 37457821 PMC10344658

[18] Fontanilla H. P. , Woodward W. A. , Lindberg M. E. , Kanke J. E. , Arora G. , Durbin R. R. , Yu T. K. , Zhang L. , Sharp H. J. , Strom E. A. , Salehpour M. , White J. , Buchholz T. A. , and Dong L. , Current Clinical Coverage of Radiation Therapy Oncology Group-Defined Target Volumes for Postmastectomy Radiation Therapy, Practical Radiation Oncology.(2012) 2, no. 3, 201–209, 10.1016/j.prro.2011.10.001, 2-s2.0-84862639582, 24674124. 24674124

[19] Goyal S. , Tiwari R. , Narayanan G. S. , and Narayanan S. S. , A Comparative Study Evaluating the Dose Volume Parameters in 3D Conformal Radiation of Left Sided Whole Breast Irradiation Including Regional Lymphnodes—A Need of Resource Constrained Countries, Reports of Practical Oncology and Radiotherapy. (2021) 26, no. 6, 1003–1009, 10.5603/RPOR.a2021.0125, 34992874. 34992874 PMC8726429

[20] White J. , Tai A. , Arthur D. , Buchholz T. , Mac Donald S. , and Marks L. , Breast Cancer Atlas for Radiation Therapy Planning: Consensus Definitions, RTOG-Radiation Therapy Oncology Group. (2016) .

[21] Ko H. , Chang J. S. , Moon J. Y. , Lee W. H. , Shah C. , Shim J. S. , Han M. C. , Baek J. G. , Park R. H. , Kim Y. B. , and Kim J. S. , Dosimetric Comparison of Radiation Techniques for Comprehensive Regional Nodal Radiation Therapy for Left-Sided Breast Cancer: A Treatment Planning Study, Frontiers in Oncology. (2021) 11, no. 11, 645328, 10.3389/fonc.2021.645328, 33912459. 33912459 PMC8072050

[22] Sabater S. , Gascon M. , Gutierrez-Perez M. , Berenguer R. , Donovan E. M. , Harris E. J. , and Arenas M. , Influence of Body Habitus on Dose Parameters of Nodal Levels III to IV Irradiation for Breast Cancer: Comparison of 3 Techniques, Medical Dosimetry. (2018) 43, no. 4, 328–333, 10.1016/j.meddos.2017.11.002, 2-s2.0-85044757418, 29223303. 29223303

